# Specific structural changes in Parkinson’s disease-related olfactory dysfunction compared to others forms of olfactory dysfunction

**DOI:** 10.3389/fncir.2024.1503841

**Published:** 2024-11-13

**Authors:** Sarah Brosse, Cécilia Tremblay, Inés Mérida, Johannes Frasnelli

**Affiliations:** ^1^Department of Anatomy, Université du Québec à Trois-Rivières, Trois-Rivières, QC, Canada; ^2^Civin Laboratory for Neuropathology, Banner Sun Health Research Institute, Sun City, AZ, United States; ^3^CERMEP-Imagerie du Vivant, Bron, France; ^4^Research Center of the Sacré Coeur Hospital of Montreal, Montreal, QC, Canada

**Keywords:** Parkinson’s disease, olfactory dysfunction, trigeminal system, gray matter, white matter, insula

## Abstract

**Context:**

Olfactory dysfunction (OD) is a common early symptom of Parkinson’s disease (PD). However, OD is not specific to PD, as approximatively 20% of the general population exhibit different forms of OD. To use olfactory measures for early Parkinson screening, it is crucial to distinguish PD-related OD from Non-Parkinsonian OD (NPOD).

**Objectives and hypothesis:**

This study aimed to compare the structural changes associated with PD-related OD (*n* = 15) with NPOD (*n* = 15), focusing on gray matter volumes and white matter fiber integrity in chemosensory regions. We hypothesized that PD-related OD presents specific structural alterations in these regions.

**Methods:**

Participants underwent a 3 T MRI scan, which included anatomical T1 and diffusion-weighted imaging. Gray and white matter integrity were assessed using both whole-brain analyses (voxel-based morphometry—VBM and tract-based spatial statistics—TBSS, respectively) and localized approaches, including regions of interest and tractography.

**Results:**

PD patients exhibited significantly higher gray matter volume in the left insula using restricted regions-of-interest analyses, while no other significant gray or white matter differences were found between groups.

**Conclusion:**

Structural imaging of the gray matter, particularly the insula, but not of white matter, differentiates PD-related OD from NPOD.

## Introduction

Olfactory dysfunction (OD) is a common symptom of Parkinson’s disease (PD), affecting over 90% of patients (Haehner et al., 2009; Doty, 2012; Fullard et al., 2017; Oppo et al., 2020; Alonso et al., 2021). OD often manifests itself in preclinical stages, years before the onset of the characteristic motor symptoms, thereby preceding the diagnostic of the disease (Berg et al., 2015). This underscores the potential of using OD as a prodromal biomarker for PD. However, OD is not specific to PD, as it affects up to 20% of the general population (Desiato et al., 2021). There are many underlying causes of non-parkinsonian OD (NPOD), such as viral infections of the upper respiratory tract, traumatic brain injury, sinonasal diseases, congenital anosmia, exposure to toxic substances, or nasal surgery (Whitcroft et al., 2023).

When aiming to use olfactory measures for early PD screening, a crucial initial step is therefore to differentiate PD-related OD from other forms of NPOD (Tremblay and Frasnelli, 2021; Orso et al., 2024). In this context, the trigeminal system a third chemosensory system alongside smell and taste, that allows the perception of sensations such as the freshness, warmth, and pungency of odorants, through the stimulation of the trigeminal nerve (Cranial Nerve V) (Terrier et al., 2022), is of particular interest (Tremblay and Frasnelli, 2021; Orso et al., 2024). This system closely interacts with the olfactory system for the perception of odorants, and this interaction appears to be affected in a disease-specific manner in PD (Tremblay et al., 2020; Tremblay and Frasnelli, 2021). In fact, in contrast to NPOD which is typically associated with a reduced trigeminal sensibility (Gudziol et al., 2001; Frasnelli et al., 2006), PD patients exhibit OD with an unimpaired trigeminal system, when measured behaviorally (Tremblay et al., 2017). Moreover, electrophysiological recordings of the nasal mucosa and functional magnetic resonance imaging (MRI) techniques suggest that PD specifically alters the central interaction between the trigeminal and the olfactory systems thus allowing the trigeminal system to maintain its integrity despite an impaired olfactory system (Tremblay et al., 2019, 2020; Tremblay and Frasnelli, 2021).

The olfactory and trigeminal systems are two independent systems that activate distinct brain areas but also share overlapping central processing areas such as (1) the insula, (2) the piriform cortex, and (3) the orbitofrontal cortex (Albrecht et al., 2010). First, the insula is crucially involved in the processing of olfactory and trigeminal stimuli (Nieuwenhuys, 2012). More specifically, while the anterior insula receives projections from the piriform cortex (Kurth et al., 2010) and is therefore considered an olfactory processing center, the medial insula receives somatosensory projections related to trigeminal information and pain (Afif et al., 2010; Kurth et al., 2010). Second, the piriform cortex is a primary olfactory processing area involved in the detection, recognition, and memory of odors, but it also responds to trigeminal activation (Gottfried et al., 2004; Zelano et al., 2007; Albrecht et al., 2010; Chevy and Klingler, 2014). The orbitofrontal cortex, finally, has reciprocal connections with the primary olfactory cortex, integrates olfactory-trigeminal information (Gottfried and Zald, 2005) and supports higher-level cognitive processes, including the perception of odor quality and experience-dependent modulation (Gottfried, 2007).

Recently, a model has been proposed to explain the hitherto unknown underlying mechanisms of alterations of olfactory-trigeminal interactions in PD-related OD (Tremblay and Frasnelli, 2021). This model stipulates that the interaction between olfactory and trigeminal central nervous areas is affected—on both, functional and structural levels—in a disease-specific manner in PD. On a functional level, this is, in fact the case: functional connectivity is (a) reduced between anterior and medial insula and (b) increased between primary olfactory and trigeminal processing areas; while this is not the case in NPOD (Tremblay et al., 2020). In addition, PD patients exhibit significantly different network modularity within the chemosensory network, when compared to NPOD patients (Tremblay et al., 2020). In summary, this supports the notion of an impaired pattern of connectivity between olfactory and trigeminal processing areas in PD, that is distinct from NPOD. However, the structural changes affecting the interaction between olfactory and trigeminal central nervous areas in PD are yet unknown. This is particularly interesting as the reliability of trigeminal testing to distinguish PD-related OD from NPOD, while being promising, is not yet fully established and its clinical usefulness in the current form remains doubtful (Orso et al., 2024). Combining neuroimaging data with behavioral tests may offer more conclusive insights (Berendse and Ponsen, 2009; Georgiopoulos et al., 2015; Tremblay et al., 2019).

The aim of this exploratory study was therefore to assess PD-specific structural changes in gray and white matter within chemosensory processing areas by comparing PD patients with NPOD patients. We focused on structural changes in olfactory processing (piriform and orbitofrontal cortex), trigeminal processing (thalamus and postcentral gyrus), and chemosensory integration (medial and anterior insula) areas. We hypothesized that PD would be characterized by preserved structural integrity in the trigeminal regions, in contrast to NPOD, where we anticipate structural alterations. Additionally, we expected that structural changes in PD would underly functional connectivity changes observed in PD but not in NPOD, suggesting potential compensatory mechanisms in PD (Tremblay et al., 2020). To evaluate gray and white matter integrity, we analyzed anatomical and diffusion MRI data and employed whole-brain analyses (voxel-based morphometry [VBM] and tract-based spatial statistics [TBSS], respectively) as well as localized approaches, including tractography and regional extraction of regions of interest.

## Materials and methods

### Participants

A total of 30 participants completed the study: 15 PD patients (age: 66.8 ± 7.3 years, 7 women, Hoehn and Yahr (H&Y) stage: 1.6 ± 0.6 (1–3), disease duration: 6.3 ± 2.8 years) and 15 matched patients diagnosed with olfactory dysfunction (age: 62.8 ± 9.2 years, 6 women, disease duration: 10 ± 9 years) either caused by a viral infection of the upper respiratory tract (postviral; *n* = 10) or sinunasal disease (sinunasal; *n* = 5). The probable cause of OD was subjectively evaluated using a questionnaire based on the position paper on OD (Hummel et al., 2017).

These participants were included in a larger MRI study, see earlier published reports (Tremblay et al., 2020; Tremblay et al., 2020). The study was conducted in accordance with the Declaration of Helsinki and approved by the local ethics committees (University of Quebec at Trois-Rivières and Research Center of the Institut Universitaire de Gériatrie de Montréal at the University of Montréal). Written informed consent was obtained from all subjects before the study.

### Neuropsychological evaluation

Olfactory function was assessed using the standardized “Sniffin Sticks” test battery (Burghart, Wedel, Germany), which includes detection threshold, odor discrimination, and identification tasks (Hummel et al., 1997). The final score (TDI) was calculated by summing the results of the three subtests (Threshold + Discrimination + Identification). Global cognitive function was evaluated using the Montreal Cognitive Assessment (MoCA) (Nasreddine et al., 2005) and depressive symptoms were measured by the Beck Depressive Inventory (BDI) (Beck et al., 1961).

*T*-test were conducted to compare TDI, MoCA and BDI scores between PD and NPOD patients.

### MRI data acquisition

All participants underwent an MRI exam on a 3.0 Tesla Prisma Fit MRI scanner (Siemens Magnetom) using a 32-channel head coil, at the Functional Neuroimaging Unit (UNF) of the research center of the Institut Universitaire de Gériatrie de Montréal. A T1-weighted 3D magnetization prepared rapid acquisition gradient echo (MPRAGE) sequence (echo time [TE]: 2.17 ms, repetition time [TR]: 2400.0 ms, flip angle: 8°, voxel size: 1 × 1 × 1 mm^3^, 176 contiguous sagittal slices, Field of view [FOV]: 224 mm) was acquired for anatomical reference. Whole-brain diffusion-weighted images were also acquired using spin-echo planar imaging (diffusion gradient directions = 108, *b*-values = 0; 300; 1,000; 2,000 s/mm^2^, FOV = 220 mm, voxel size = 2 × 2 × 2 mm^3^). PD patients were in their usual dopaminergic medication state during MRI scanning to control for involuntary head motions. The MRI session included both structural and functional scans; results on functional connectivity within the chemosensory system are published elsewhere (Tremblay et al., 2020).

### MRI data preprocessing

The preprocessing was performed with the support provided by Calcul Québec^1^ and the Alliance.^2^ DICOM files were first converted to NIFTI format. Diffusion MRI data were corrected for distortions induced by eddy currents and magnetic susceptibility as well as subject motion using FSL (EDDY and TOPUP toolboxes) (Andersson and Sotiropoulos, 2016). Fractional anisotropy (FA) and mean diffusivity (MD) maps were generated for each participant using the DTIFIT tool implemented in FSL. Briefly, FA reflects the directionality of water diffusion in tissue, while MD represents the average rate of diffusion. Lower FA values indicate disrupted or less organized white matter structures, whereas higher MD values are typically associated with increased water movement, often linked to tissue damage or degeneration.

### Anatomical MRI segmentation

The structural 3D T1-weighted images were automatically segmented into anatomical regions using the multi-atlas propagation with enhanced registration (MAPER) method (Heckemann et al., 2010) and the 120-region Hammersmith atlas (Hammers et al., 2003; Gousias et al., 2008; Steinbart et al., 2023). White matter and gray matter probability maps obtained with the segment function (Statistical Parametric Mapping [SPM12]) were thresholded at 0.5 and combined with the 120-ROI anatomical segmentation in order to separate their gray and white matter parts, expect for pure white matter regions such as the corpus callosum, and pure gray matter regions such as the basal ganglia.

### Voxel-based morphometry

To explore gray matter volume across our two groups of participants, voxel-based morphometry (VBM) analysis was performed using the standard VBM processing protocol of the Computational Anatomy Toolbox (CAT12) running on SPM12. First, 3D T1-weighted images were segmented into gray matter, white matter and cerebrospinal fluid tissue classes using SPM’s unified segmentation function. We assessed group differences in gray matter volume using a two-tailed *t*-test in CAT12 with TDI, age and sex as covariables. We performed a restricted analysis applying an explicit Activation Likelihood Estimation (ALE) derived mask composed of regions functionally activated by intranasal trigeminal stimulation with carbon dioxide (CO_2_) (Albrecht et al., 2010) and regions functionally activated by olfactory stimulation (Torske et al., 2022).

### Gray matter regional extraction

**Figure 1:**
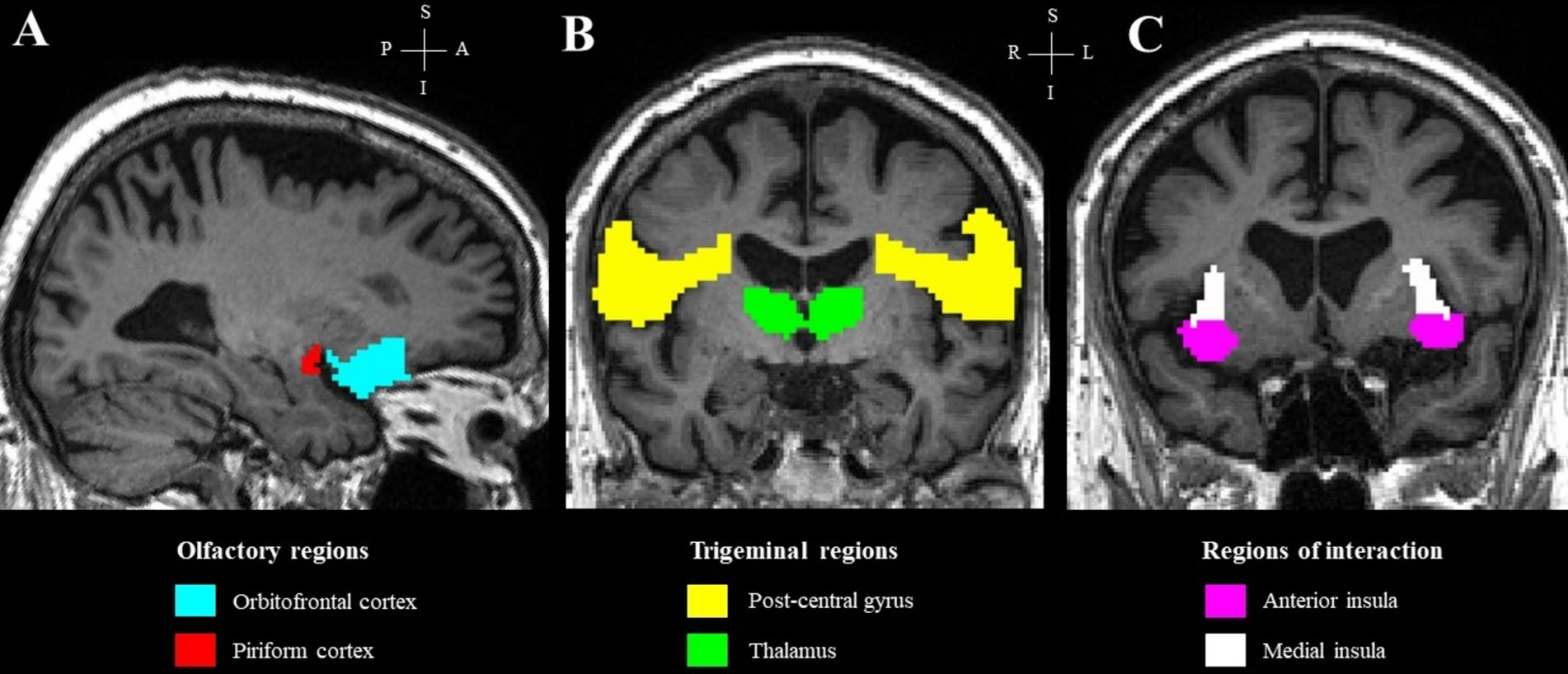
Regions of interest in a representative subject, overlaid on the subject’s T1 image. **(A)** Olfactory regions; **(B)** trigeminal regions; **(C)** regions of interaction.

To further explore potential group differences in specific chemosensory regions of interest, gray matter volumes were extracted at defined ROIs and compared between the two groups of participants using RStudio (2024.04.1 version). The selected ROIs included the orbitofrontal cortex, piriform cortex, post-central gyrus, thalamus, and the anterior and medial insula to specifically represent the olfactory and trigeminal regions, as well as the regions of interaction between the two systems (Figure 1). These regions were defined using masks derived from anatomical segmentation of T1-weighted MRI, as described previously (Hammers et al., 2003; Gousias et al., 2008; Steinbart et al., 2023).

### White matter integrity analysis

Local FA and MD differences between groups were mapped at the voxel level with TBSS (Smith et al., 2006) implemented in FSL. A general linear model analysis including unpaired Student’s *t*-test (PD patients vs. NPOD patients) with TDI, age and sex as covariates, was used to compare FA and MD between groups. Results were corrected for multiple comparisons using family-wise error (FWE) correction and threshold-free cluster enhancement.

**Figure 2:**
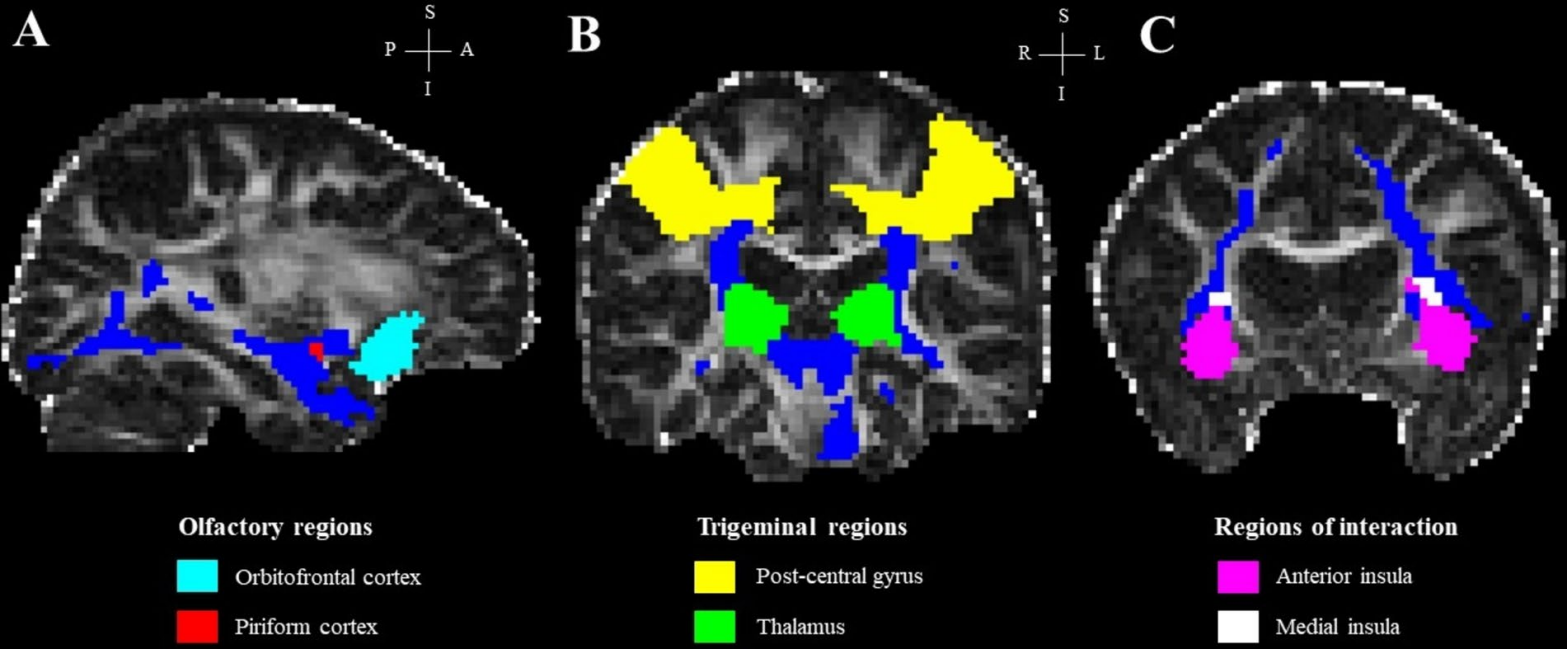
Regions of interest and final tracts in a representative subject, overlaid on the subject’s FA image. **(A)** Olfactory regions; **(B)** trigeminal regions; **(C)** regions of interaction. Blue streamlines in panels **(A–C)** represent the final tract for each seed-target pair.

In addition, the integrity of white matter fibers connecting our ROIs, which were defined using masks derived from the anatomical segmentation of the T1-weighted MRI (Hammers et al., 2003; Gousias et al., 2008; Steinbart et al., 2023), was investigated by tractometry. The piriform cortex, medial insula and thalamus were used as seed regions and the orbito-frontal cortex, anterior insula and the post-central gyrus were, respectively, used as target regions. First, fiber orientations were estimated in each voxel using BEDPOSTX (FSL). Then, probabilistic tractography was performed using PROBTRACKX (FSL) with default settings (5,000 streamlines/voxel). An exclusion mask for the contralateral hemisphere was specified for each seed-target pair. The strength and the most likely location of a pathway between the seed and its target region was calculated (fdt_path). Tractography results (fdt_path) for each seed-target pair were divided by the waytotal number associated (a measure of the total number of streamlines between each seed-target pair) and thresholded to 0.007 to normalize the tracts (Hecht et al., 2015). Finally, the resulting tracts were binarized and used as masks to extract mean FA and MD values for each participant (Figure 2). For each seed-target pair, the unequal variance *t*-test was performed to examine the difference in regional FA and MD values, as well as the waytotal number, between PD and NPOD patients using RStudio, with the TDI score included as a covariate.

For all analyses, a family-wise error (FWE) correction with a *p*-value inferior to 0.05 or, in a second step, an uncorrected *p*-value <0.001 for predicted areas, was considered statistically significance.

## Results

### Neuropsychological evaluation

There were no significant age differences between the two groups [*t*(27) = 1.32; *p =* 0.20]. A *t*-test comparing the TDI scores between PD patients (17.5 ± 6.9) and NPOD (17.3 ± 7.7) revealed no significant difference in olfactory function between the two groups [*t*(27) = 0.07; *p =* 0.94], and both groups’ averages were in the range of hyposmia (Oleszkiewicz et al., 2019). No difference in global cognitive function, as measured by the MoCA, was observed between PD patients (27.0 ± 2.8) and NPOD patients (27.2 ± 2.3) [*t*(27) = −0.21; *p =* 0.83]. However, a significant difference in depressive symptoms was found, as measured BDI [*t*(27) = 3.29; *p =* 0.003], with PD patients (6.1 ± 3.4) showing significantly higher levels of depression compared to NPOD patients (2.3 ± 2.9).

### Gray matter integrity

#### VBM

To explore gray matter volume differences between the two groups, we employed a two-step approach. First, a whole-brain VBM analysis was performed using the standard VBM processing protocol, which revealed no significant differences between PD and NPOD patients (FWE, *p* < 0.05). Next, we focused the VBM analysis on regions activated by trigeminal and olfactory stimulation. In this restricted analysis, PD patients showed higher gray matter volume (*p* < 0.001, uncorrected) in the left insula and the cerebellar vermis compared with NPOD patients (Table 1). No significant differences were found for the contrast PD < NPOD.

**Table 1:** Statistical parametric mapping results of the two samples *t*-test on gray matter volume (uncorrected, *p* < 0.001).

Contrast	Region	Side	*t*-value	Cluster size	Peak MNI coordinate (*X*, *Y*, *Z*)
NPOD<PD	Insula	L	3.81	26	−34, −2, −12
	Cerebellar vermis	–	3.66	5	−8, −56, −28

L, left; MNI, Montreal Neurological Institute.

#### ROI

PD patients exhibited significantly higher gray matter volume in the anterior insula compared to NPOD patients (Table 2). No difference was found in the other ROIs (Table 2).

**Table 2:** Region-of-interest analysis of the gray matter volume of PD patients and patients with NPOD.

	ROI	PD	NPOD	*p* Value	*t*-Value
		Mean voxel number	Mean voxel number		
Olfactory regions	Orbitofrontal cortex	12,374	12,386	0.99	−0.02
	Piriform cortex	437	436	0.96	0.05
Trigeminal regions	Thalamus	4,972	4,778	0.61	0.52
	Postcentral gyrus	10,870	10,354	0.40	0.86
Interaction regions	Anterior insula	2,585	2,314	0.04*	2.21
	Medial insula	1,144	1,047	0.17	1.42

*p < 0.05. Statistically significant results are highlighted in bold.

### White matter integrity

#### TBSS

There were no significant differences in the diffusion imaging indices (FA and MD) between PD patients and NPOD patients (FWE, *p* < 0.05) at voxel-wise level.

#### Tractography

There were no regional significant differences in the diffusion imaging indices (FA and MD) and the waytotal number between PD patients and NPOD patients (Table 3).

**Table 3:** Region-of-interest analysis of diffusion tensor imaging indices in the white matter of PD patients and NPOD patients.

	Seed → target	Metric	PD	NPOD	*p* Value	*t*-Value
			Mean	Mean		
Olfactory regions	Piriform cortex → Orbitofrontal cortex	FA	0.39	0.40	0.57	−0.58
		MD	6.79 × 10^−4^	6.67 × 10^−4^	0.30	1.06
		Waytotal	32,055	35,165	0.61	−0.52
Trigeminal regions	Thalamus → Postcentral gyrus	FA	0.46	0.45	0.49	0.70
		MD	6.13 × 10^−4^	6.11 × 10^−4^	0.84	0.20
		Waytotal	570,205	443,344	0.21	1.30
Interaction regions	Medial insula → Anterior insula	FA	0.40	0.39	0.44	0.79
		MD	6.17 × 10^−4^	6.17 × 10^−4^	0.98	0.03
		Waytotal	419,711	391,105	0.13	1.55

PD, Parkinson’s disease; NPOD, non parkinsonian olfactory dysfunction; FA, fractional anisotropy; MD, mean diffusivity.

## Discussion

In the present study, we investigated structural changes in gray and white matter within chemosensory areas in PD patients compared to NPOD patients. Our main finding is that PD patients exhibited significantly higher gray matter volume in the left insula. This increase of gray matter in the insula was detected in both restricted analysis of regions of interest (VBM analysis on trigeminal and olfactory regions and ROI extraction analysis). We did not observe any other alterations in gray or white matter between the groups.

### Structural alterations in PD and NPOD patients

Previous research shows that both PD and NPOD are associated with structural changes in regions related to olfaction in both gray and white matter. With regards to (1) PD, these changes are characterized by decreased FA and increased MD in white matter areas involved in olfactory processing, including the orbitofrontal cortex, entorhinal cortex, cerebellum, and olfactory sulcus (Ibarretxe-Bilbao et al., 2010; Zhang et al., 2011; Georgiopoulos et al., 2017). In early-stage PD, reduced FA in white matter adjacent to primary olfactory regions and the gyrus rectus has been correlated with olfactory dysfunction (Ibarretxe-Bilbao et al., 2010). A meta-analysis further indicated decreased FA in the right olfactory cortex among medication-free PD patients (Suo et al., 2021). Additionally, advanced PD patients show increased axial, mean, and radial diffusivity in olfactory-related white matter regions (Hummel et al., 2021). Surprisingly, one study reported decreased FA values in the olfactory tract in PD patients compared to controls, but also observed increased FA values in several other brain regions, including the corticospinal tract, superior longitudinal fasciculus and cingulum (Chen et al., 2018). PD patients also show decreased gray matter volume in brain areas associated with olfactory functions, such as the olfactory bulb, piriform cortex, amygdala, and entorhinal cortex (Wattendorf et al., 2009; Chen et al., 2014; Li et al., 2016). The correlation between olfactory scores and gray matter atrophy is significantly higher in PD patients compared to controls (Wattendorf et al., 2009). In summary, PD appears to be associated with both gray and white matter alterations in key olfactory processing areas.

Similarly, (2) NPOD is also associated with structural changes in regions related to olfaction in both gray and white matter, with alterations observed in patients with both anosmia (complete loss of smell) and hyposmia (reduced sense of smell) (Bitter et al., 2010; Gao et al., 2022; Hura et al., 2022). A recent systematic review reported reduced gray matter volume in NPOD patients in the orbitofrontal cortex, anterior cingulate cortex, insular cortex, parahippocampal cortex, piriform cortex, cerebellum, fusiform cortex, middle temporal gyrus, and middle frontal gyrus/cortex (Hura et al., 2022). Additionally, reduced white matter volume was found surrounding the anatomical atrophy of these gray matter regions (Hura et al., 2022). Conversely, no study has reported an increase in gray matter and/or white matter volume in NPOD patients compared to controls (Hura et al., 2022). In summary, NPOD is associated with widespread reductions in both gray and white matter volumes in olfactory-related regions, without evidence of compensatory increases in these areas.

The insula, a region involved in olfactory and trigeminal interaction, has previously shown changes in functional connectivity in PD, in the same cohort (Tremblay et al., 2020). Our results may be interpreted as compensatory mechanisms in PD, absent in NPOD. While these findings suggest potential insula-specific changes in PD, the limited statistical power and the absence of other structural imaging studies directly comparing PD and NPOD patients, this interpretation has to be taken with caution. Futures studies with larger sample sizes should further explore insula-related changes in PD.

The PD-specific alteration of the trigeminal-olfactory interaction being a relatively recent finding, no study has specifically investigated structural effects of PD on trigeminal processing areas. In turn, NPOD is characterized by a reduction of gray matter in trigeminal processing regions, such as the thalamus and the insula (Bitter et al., 2010; Hura et al., 2022).

This review of the literature shows that both PD and NPOD lead to structural alterations in gray and white matter of chemosensory processing areas. When directly comparing both groups, no differences in olfactory bulb volumes could be found, although machine learning approaches suggested that the surrounding olfactory bulb cortical areas allowed for a discrimination between the two conditions (Tremblay et al., 2020). We extend these observations by showing increased gray matter in PD patients in the left insula through restricted regions-of-interest analyses, and in the cerebellar vermis through VBM restricted analysis, but no differences in white matter integrity were observed. This may suggest that observed structural changes in chemosensory areas in PD may be associated with olfactory dysfunction, rather than being specific to PD. This underlines the necessity to include NPOD patients as controls in future studies.

### Functional vs. structural alterations

Recent studies indicate specific functional alterations in chemosensory regions of PD patients compared to NPOD patients (Tremblay et al., 2020; Georgiopoulos et al., 2024). Specifically, PD patients exhibit impaired functional connectivity between olfactory and trigeminal processing areas, with particular focus on the connexions between the anterior and medial insula, as well as the piriform cortex (Tremblay et al., 2020; Georgiopoulos et al., 2024). Additionally, abnormal connexions between gray matter areas related to olfaction and white matter fiber bundles have been identified in PD patients compared to controls (Du et al., 2022).

To better characterize the olfactory and trigeminal systems and their interaction in PD, one of the objectives of this study was to investigate whether the previously observed functional alterations (related to BOLD changes during a task) were supported by structural differences (e.g., size or shape) in gray and white matter. This is important since combining various imaging modalities has proven beneficial in classification of neurodegenerative diseases. For example, combining information from anatomical MRI, diffusion weighted MRI, and resting state functional MRI can improve Alzheimer disease classification (Schouten et al., 2016). Nevertheless, our study did not reveal any structural differences in the chemosensory regions between PD and NPOD patients—except for the insula as previously discussed—even though functional differences were observed in the same cohort (Tremblay et al., 2020). The relationship between structural and functional alterations is complex. For example, disrupted structural connectivity may be associated by increased functional activity as a compensatory mechanism (Zhou et al., 2020). In early PD, functional connectivity changes precede structural connectivity changes (Meles et al., 2021). Our study suggests that, in distinguishing PD patients from NPOD patients through the assessment of olfactory and trigeminal senses, functional imaging appears to be more suitable than structural imaging. Further investigation using EEG, which provides high temporal precision, would be valuable. Combining multiple functional techniques is essential for better understanding the central interactions between the trigeminal and olfactory systems in PD compared to NPOD.

### Limitations

This exploratory study aimed to characterize the structural integrity of chemosensory regions in PD compared to NPOD, following the observation of functional differences in the same cohort of participants (Tremblay et al., 2020). We acknowledge that the sample size used in this study is relatively small; hence, the results should be interpreted carefully. This may also explain the relatively few differences in gray matter volumes and the absence of differences in white matter integrity between groups. However, PD patients were carefully matched to a non-PD group with a similar level of olfactory impairment to identify underlying mechanism specific to PD-related OD. Moreover, participants completed a behavioral test to assess their olfactory function (TDI), but no test was conducted to evaluate their trigeminal system. While it would be valuable to include an assessment of the trigeminal system in future studies, available trigeminal tests are both time-consuming and lack precision (Hummel and Kobal, 1999; Hummel et al., 2003). Therefore, the development of practical tools to measure trigeminal sensitivity is of primary importance (Hummel et al., 2016; Huart et al., 2019; Jobin et al., 2021). Future studies could enhance our approach by including axial and radial diffusivity values into the analyses, providing additional insights into white matter alterations.

## Conclusion

In summary, we are presenting preliminary findings regarding the structural alterations in chemosensory areas between PD and NPOD patients. While we observed increased gray matter volume in the left insula of PD patients, no other significant differences in gray or white matter were found between the two groups. These results suggest that structural changes in chemosensory regions may be more related to olfactory dysfunction in general rather than being specific to PD. Furthermore, our findings indicate that functional imaging may be more effective for differentiating PD from NPOD by capturing the altered interaction between olfactory and trigeminal systems.

## Data Availability

The raw data supporting the conclusions of this article will be made available by the authors without undue reservation.

## Footnotes

^1^ calculquebec.ca

^2^ alliancecan.ca/en
